# Implementation of the CMOS MEMS Condenser Microphone with Corrugated Metal Diaphragm and Silicon Back-Plate

**DOI:** 10.3390/s110606257

**Published:** 2011-06-10

**Authors:** Chien-Hsin Huang, Chien-Hsing Lee, Tsung-Min Hsieh, Li-Chi Tsao, Shaoyi Wu, Jhyy-Cheng Liou, Ming-Yi Wang, Li-Che Chen, Ming-Chuen Yip, Weileun Fang

**Affiliations:** 1 Department of Power Mechanical Engineering, National Tsing Hua University (NTHU), Hsinchu 30013, Taiwan; E-Mails: d947708@oz.nthu.edu.tw (C.-H.H.); mcyip@pme.nthu.edu.tw (M.-C.Y.); 2 Department of Microelectromechanical Systems, United Microelectronics Corporation (UMC), Hsinchu 30078, Taiwan; E-Mails: Ming_Yi_Wang@umc.com (M.-Y.W.); Anchor_Chen@umc.com (L.-C.C.); 3 Department of Device Development, Solid State System Corporation (3S), Hsinchu 30288, Taiwan; E-Mails: jason_lee@3system.com.tw (C.-H.L.); tmhsieh@3system.com.tw (T.-M.H.); lichi_tsao@3system.com.tw (L.-C.T.); shaoyi@3system.com.tw (S.W.); chengliou@3system.com.tw (J.-C.L.)

**Keywords:** CMOS-MEMS, condenser microphone, corrugated, sensitivity, diaphragm

## Abstract

This study reports a CMOS-MEMS condenser microphone implemented using the standard thin film stacking of 0.35 μm UMC CMOS 3.3/5.0 V logic process, and followed by post-CMOS micromachining steps without introducing any special materials. The corrugated diaphragm for the microphone is designed and implemented using the metal layer to reduce the influence of thin film residual stresses. Moreover, a silicon substrate is employed to increase the stiffness of the back-plate. Measurements show the sensitivity of microphone is −42 ± 3 dBV/Pa at 1 kHz (the reference sound-level is 94 dB) under 6 V pumping voltage, the frequency response is 100 Hz–10 kHz, and the S/N ratio >55 dB. It also has low power consumption of less than 200 μA, and low distortion of less than 1% (referred to 100 dB).

## 1. Introduction

Presently, microphones have become an important component in various portable consumer electronics, including cellular phones, personal digital assistants and computers. In these applications, size and cost are two critical concerns for microphone design. Silicon based MEMS (Micro electro mechanical systems) microphones have the advantages of offering small size, low cost, and ease of integration with CMOS (Complementary Metal Oxide Semiconductor) circuits. Moreover, the MEMS microphone is also compatible with the surface mount process [1]. There are various design considerations for MEMS microphone such as less interference, better sound quality, higher sensitivity, higher signal to noise ratio, less current drain, and low distortion. Nevertheless, the fundamental challenge of MEMS microphone remains the availability of a reliable fabrication processes to implement the devices. Various small size and low cost microphones realized by different MEMS technologies have been reported in [2,3]. The well-known two poly-Si surface micromachining process has been exploited to implement commercial microphones [4]. Such microphone design consists of a spring and diaphragm made of poly-Si thin films.

The fabrication of MEMS structures and components using available CMOS foundries is a promising approach to implement micro sensors. Presently, the standard CMOS processes together with the post-CMOS processes have been employed extensively to realize MEMS devices [5]. Thus, the monolithic integration of IC and MEMS components can be easily achieved. In this regard, the available mature foundry CMOS process is considered as a promising approach to implement the MEMS microphone; and the MEMS microphone and its signal processing IC can thus be monolithically integrated on a single chip. In this way, the parasitic capacitance can be reduced to enhance the signal-to-noise ratio of a condenser microphone. The IC/MEMS monolithic integrated condenser microphone implemented on an available CMOS foundry has been demonstrated in Akustica [5]. However, the deposition of polymer film is required for the diaphragm of the microphone. This is not a standard material for CMOS process.

In general, the CMOS MEMS microphone design parameters, such as the shape and dimensions of the diaphragm, back-plate, and air gap are limited by the standard CMOS process. Thus, the performance of a CMOS MEMS microphone is also restricted by the process. This study presents the design and implementation of a novel CMOS MEMS condenser microphone based on the standard UMC (United Microelectronics Corp., Taiwan) CMOS process on 8-in wafers. In addition, the post-CMOS bulk and surface micromachining processes are also performed in the foundry. Such a condenser microphone consists of a corrugated metal diaphragm [6–8] and a thick and stiff silicon back-plate to improve its performance. Measurements indicate the sensitivity of microphone is −42 ± 3 dBV/Pa at 1 kHz (the reference sound-level is 94 dB) under 6 V pumping voltage, the frequency response is 100 Hz–10 kHz, and the *S/N* ratio >55 dB.

## 2. Concepts and Design

**Figure 1.**
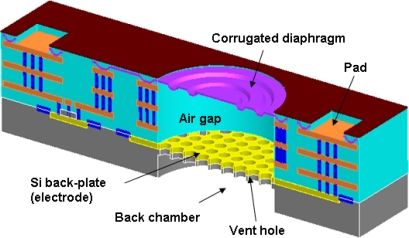
The proposed CMOS MEMS microphone design.

Figure 1 shows a schematic illustration of the proposed CMOS-MEMS capacitive microphone which consists of the diaphragm, air gap, silicon back-plate, vent holes, back chamber, and sensing circuits. The movable electrode on the diaphragm and the stationary electrode on the back-plate form a parallel capacitor. The sound pressure on the microphone will cause the deformation of the flexible diaphragm. The deformation of the rigid back-plate resulting from the sound pressure can be ignored and thus, the air gap between diaphragm and the back-plate is changed by the sound pressure and this leads to a capacitance change in the parallel capacitor. In this regard, the corrugated diaphragm is designed to reduce its stiffness and also release the thin film residual stresses [6–8]. Moreover, the thick back-plate is designed to increase its stiffness. The deformation of the stationary electrode by the sound pressure can be prevented. The vent holes are also prepared on the thick back-plate. A large number of vent holes are designed in the back-plate to achieve a low streaming resistance in the air gap, which can prevent the reduction of mechanical sensitivity at higher frequencies. In addition, the existence of vent holes will also reduce the area of stationary sensing electrode.

**Figure 2.**
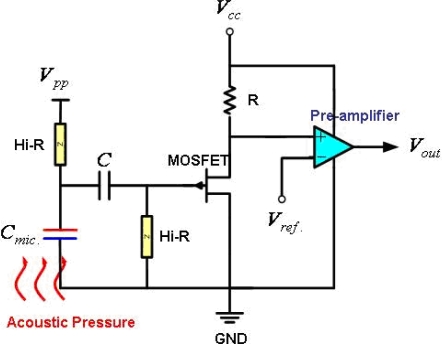
Schematic of the amplifier circuit of the fabricated condenser microphones.

**Figure 3.**
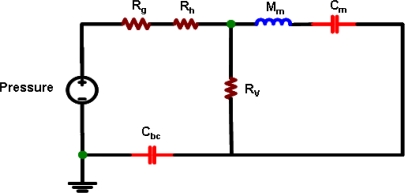
Equivalent electrical circuit analysis of the condenser microphone for PSPICE.

The device is implemented by using the standard UMC 0.35 μm 1-poly 4-metals CMOS 3.3/5.0 V logic process. Thus, it is necessary to consider the design rules, available thin film layers, and the dimensions and stacking of these layers for the process. Briefly, this standard process has a total of four metal layers, named metal-1 to metal-4. The metal-4 layer is employed to fabricate the diaphragm. The corrugated diaphragm structure is designed to release the residual stress of the metal film. Note that corrugated metal diaphragm with such a large area violates the design rules of the standard UMC process. In short, the area of diaphragm is larger than the planar dimensions of the metal layer allowed in the design rules. Nevertheless, this corrugated structure design remains acceptable for the foundry. The air gap is defined by the thickness of the thin films between the metal-4 layer and the silicon substrate. The silicon substrate is used to increase the thickness and stiffness of the back-plate, so as to prevent its deformation caused by the sound pressure. As indicated in Figure 1, the amplifier circuit is monolithically integrated with the microphone. Figure 2 further shows the amplifier circuit design. The capacitive microphone is charged by an external pumping voltage *V_pp_* from *V_cc_*. As the acoustic pressure is introduced to the microphone, the vibration of the diaphragm will cause a capacitance change between the diaphragm and back-plate. The key parameters of the microphone design and simulation are summarized in Table 1. Moreover, the equivalent circuit for the proposed MEMS microphone is shown in Figure 3. The diaphragm, air gap, vent hole, back-plate, and back chamber are described by the complex damping, mass, compliance system, and assembled in an acoustical circuit [9–12]. This equivalent small signal circuit containing the acoustic, mechanical, and electrical component can well describe the behaviors of the MEMS microphone by PSPICE. For instance, as indicated in Figure 3, the *R_g_* [kg/s], *R_h_* [kg/s] and *R_v_* [kg/s] are represented as acoustical resistance of air gap, vent holes and ventilation on diaphragm, respectively. The *C_m_* [m/N] and *M_m_* [kg] stand for mass and compliance of the diaphragm, respectively, and the *C_bc_* [m/N] represents the compliance of the back chamber.

## 3. Fabrication and Results

**Figure 4.**
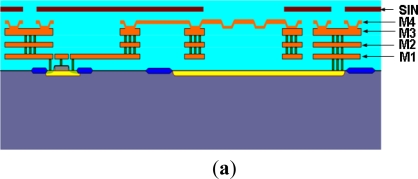

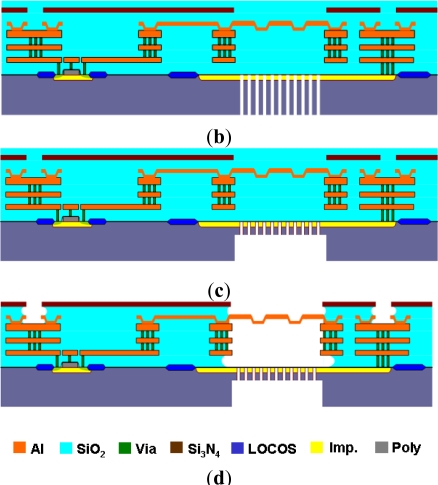
(**a**) Front-side UMC 0.35 μm 1P4M standard CMOS process; (**b**) Back-side grinding and vent hole DRIE; (**c**) Back chamber DRIE; (**d**) Microphone sacrificial layer etching.

Figure 4 shows the process steps used to fabricate the CMOS MEMS microphone in the commercial foundry of UMC on an 8-in wafer. The processes consist of the standard UMC 0.35 μm 1-poly 4-metals 3.3 V/5.0 V logic device process and the post-CMOS MEMS structure releasing process. Figure 4(a) indicates the layers stacking prepared by the UMC CMOS process. In this process, there were totally four metal films (metal-1 to metal-4). During the CMOS process, the thermal budget and thin film residual stresses should be considered so as to not to affect the device’s performance. For example, the passivation layer in the standard UMC CMOS process usually leads to residual stress problems. Such residual stresses could be released by a annealing process. However, the annealing time of the passivation layer was critical to the performance of the device. The mechanical characteristics of the microphone could shift and the sensing circuit could fail if the annealing time was not properly controlled. After removing the tungsten vias between the metal-3 and metal 4 layers, the near 2 μm thick steps were formed on the dielectric film on top of the metal-3 layer. Thus the corrugated structure was implemented on the metal-4 film to meet the design requirement for the microphone diaphragm. Such a corrugated structure also has the potential to prevent the stiction between the diaphragm and back-plate, as the air gap is reduced. The other three metal layers (*i.e.*, metal-1 to metal-3) and tungsten vias were used as the electrical routings. Moreover, some of the dielectric films were employed as the sacrificial layers, and were removed to release the suspended mechanical diaphragm.

After completing the CMOS process, the backside of the silicon substrate was thinned by a grinding process, and then etched by DRIE (Deep Reactive Ion Etch), as shown in Figure 4(b). Thus, the vent holes of the microphone were defined. In order to avoid the deformation due to the sound pressure in the back-plate, silicon was introduced into the back-plate to enhance the structure thickness as well as stiffness. As shown in Figure 4(c), the 2nd DRIE was used to fabricate the back chamber and also define the thickness of the back-plate [13]. During the backside etching processes, the oxide was deposited and patterned as the mask for the 2nd DRIE, and then covered with the patterned photoresist as the mask for the 1st DRIE. The photoresist mask was removed after the 1st DRIE, and then the substrate was patterned by the 2nd DRIE using the oxide mask [14]. Finally, the sacrificial dielectric layers were removed by wet etching to release the diaphragm from the silicon substrate, as illustrated in Figure 4(d). In this process, the Si_3_N_4_ was used as the protection layer during wet etching.

**Figure 5.**
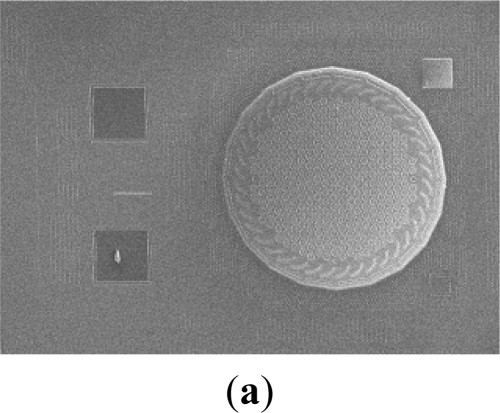

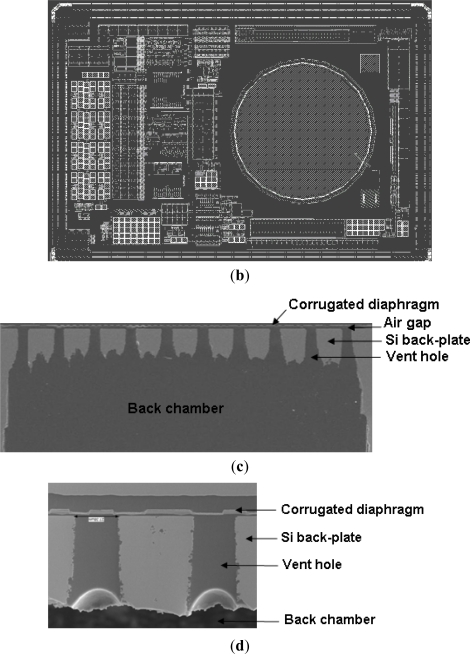
(**a**–**b**) The top view SEM micrograph of a typical fabricated CMOS MEMS microphone and its associated CMOS circuit and **MEMS** layout; (**c**) the side view SEM micrograph of a fabricated microphone; and (**d**) the zoom-in side view micrograph to show the corrugated structure and the sensing gap.

The Scanning Electron Microscope (SEM) micrograph in Figure 5(a) shows the top view of a typical as fabricated microphone chip. The diaphragm of 800 μm diameter and the bonding pads are observed. In comparison, Figure 5(b) shows the associated layout of the CMOS circuit and MEMS structure for the chip in Figure 5(a). It also indicates the monolithic integration of the mechanical structure and the signal processing circuits of the CMOS MEMS microphone. The SEM micrograph in Figure 5(c) shows the cross section of a typical fabricated microphone. The thin corrugated diaphragm, air gap, thick back-plate with vent holes, and backside chamber of the microphone are observed. The zoom-in micrograph in Figure 5(d) further demonstrates the corrugated structure and the sensing gap. The vent hole has a diameter of near 20 μm and the back-plate thickness is near 40 μm due to the existing of silicon substrate. Thus, the stiffness of back-plate is five orders of magnitude higher than that of the diaphragm.

## 4. Measurements

**Figure 6.**
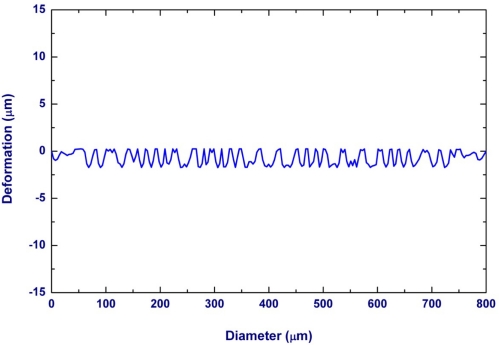
The surface profile of corrugated diaphragm along the diameter measured by the optical interferometer.

**Figure 7.**
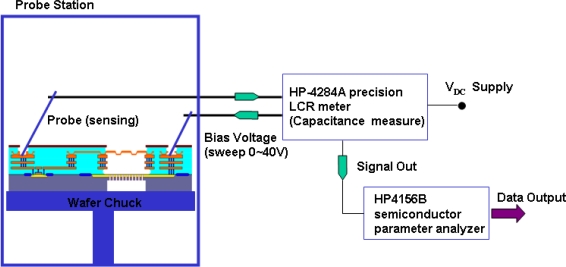
The test setup for C-V characterization.

**Figure 8.**
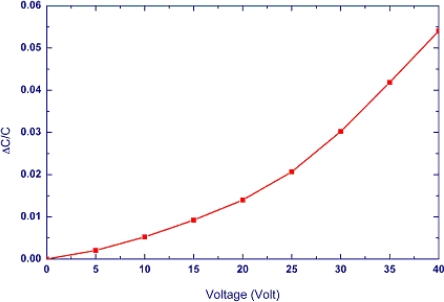
Measured capacitance sensitivity of a typical fabricated microphone.

The characteristics of the microphone have been characterized by the optical interferometer, DC voltage driving test, and sensitivity testing by the pulse electro-acoustics in an anechoic box. The optical interferometer was employed to measure the shape of microphone diaphragm. Figure 6 shows a typical measurement result. The measurement clearly displays the diaphragm structure with a 2 μm corrugation height and the initial bending curvature due to the thin film residual stresses. It indicates the bending of the microphone diaphragm has a radius of curvature of 32 m. The microphone was characterized using the voltage driving test. Figure 7 shows the test setup for C-V measurement which contains a probe station, the precision LCR meter (HP-4284A), and the semiconductor parameter analyzer (HP4156B). In this experiment, a driving DC voltage was applied on the microphone by the LCR meter. The diaphragm was then deformed by the electrostatic force induced from the DC voltage. Thus, the capacitance change of microphone caused by the diaphragm deformation was detected by the LCR meter. The typical C-V measurement results in Figure 8 show the capacitance change of microphone at different driving voltages. In addition, the microphone exhibits an initial capacitance of 1.48 pF at zero bias.

**Figure 9.**
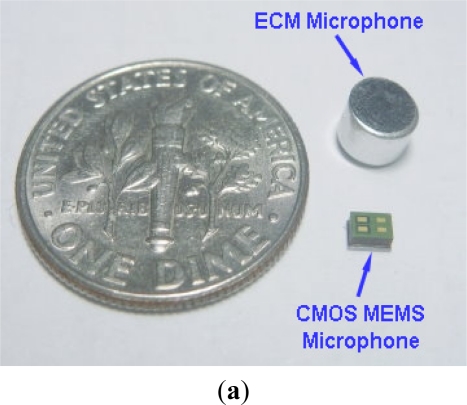

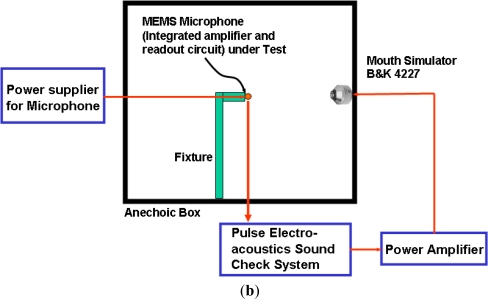
(**a**) The microphone after packaging; and (**b**) the setup of microphone sensitivity test.

**Figure 10.**
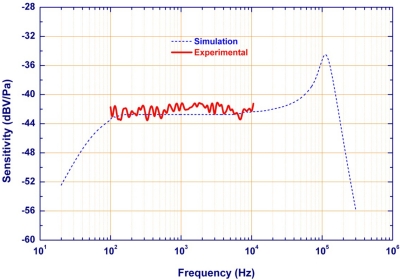
The predicted and measured frequency responses of the presented MEMS microphone.

**Figure 11.**
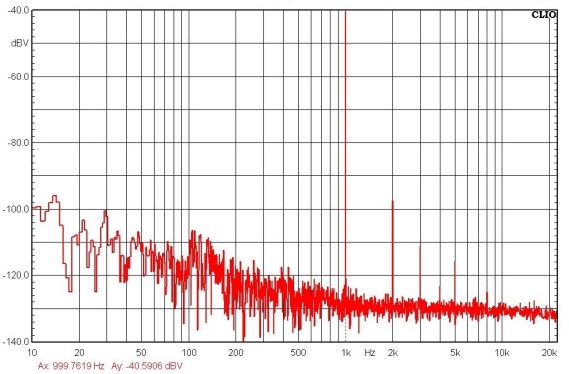
The typical measured frequency response of the fabricated microphone.

The microphone was packaged as shown in Figure 9(a), and then sensitivity tests were performed by pulse electro-acoustics in an anechoic box. The measurement setup for the sensitivity test is schematically illustrated in Figure 9(b). The microphone was placed inside a anechoic chamber for testing. The external noise and sound reflections are minimized by the anechoic chamber. The mouth simulator (B&K 4227) was used to specify a sound pressure to excite the microphone during the tests. The sound pressure level was monitored by a commercial electro-acoustics SoundCheck system (AmpConnect^™^). Measurements show the typical sensitivity of microphone is −42 dBV/Pa at 1 kHz and the bandwidth is larger than 10 kHz, as indicated in Figure 10. For comparison, the simulation results predicted using the equivalent circuit model in Figure 3 are also available in Figure 10. As shown in Figure 11, the sensitivity of the microphone is −42 dBV/Pa at 1 kHz (the reference sound-level is 94 dB). The simulations agree well with the measurements. The microphone also has a preliminary *S/N* ratio of >55 dB. Detailed measured specifications are summarized in Table 2.

## 5. Conclusions

This study presents the design of a CMOS-MEMS microphone consisting of a corrugated diaphragm that is 800 μm in diameter, and having a rigid thick back-plate mainly formed by the silicon substrate. Moreover, the tests demonstrate the performance features of the device such as sensitivity and frequency range. The microphone has flat frequency response from 100 Hz to 10 kHz, and sensitivity of −42 ± 3 dBV/Pa at 1 kHz (the reference sound-level is 94 dB). Moreover, the microphone has current consumption of less than 200 μA, the S/N ratio of over 55 dB, and low distortion of <1% (refer to 100 dB). In short, this study demonstrates the possibility of implementing the CMOS MEMS microphone by establishing and integrating the standard CMOS and post-CMOS release processes in an open CMOS foundry. This could be a critical step for the mass production of MEMS devices in such a CMOS foundry.

Nevertheless, the integration of the proposed MEMS microphone with the circuits by the standard CMOS process on 8-in wafer it is not straightforward. The design of MEMS devices is limited by the features of the standard CMOS process. Moreover, various problems and limitations should be considered by using the standard CMOS process: (1) the materials available for MEMS structures are limited to the thin films for backend processes (*i.e.*, metal 1∼4, dielectric, and tungsten); (2) Wafer warpage will cause handling problems during backend processing; (3) large metal patterns could lead to electrical arcing during via etching and cause high temperature damage to the wafer; (4) the thermal budget of any post-CMOS processing has to be considered to avoid electrical shift of the MOSFETs; and (5) metal hard masks should be prevented during sacrificial layer etching to reduce the parasitic capacitance. In addition, due to the existence of metal film structures, the creep of these metal film structures and the performance drift due the residual stresses would be critical concerns for the presented microphone. It is of importance to investigate these factors for the application of the presented microphone.

## Figures and Tables

**Table 1.** The important design parameters and dimensions of the microphone.

Diaphragm diameter	800 μm	Air hole diameter	20 μm
Diaphragm density	2,500 kg/m^3^	Air hole quantity	350
Diaphragm thickness	1.1 μm	Hole ratio	22%
Young’s module of diaphragm	21.2 GPa	Air density	1.8 kg/m^3^
Diaphragm stress	41.63 MPa	Viscosity of air	1.73 × 10^−5^ N-s/m^2^
Effective diameter of backplate	800 μm	Bias voltage	6 V
Backplate thickness	40 μm	Parasitic capacitor	0.7 pF
Initial air gap	4.2 μm		

**Table 2.** Measured specifications of the packaged microphone.

Size L × W × H	2.35 × 1.65 × 1.2 mm^3^	Out impedance	<350 Ω
Operation voltage	1.65–3.6 V	PSRR	−60 dB
Sensitivity (1 kHz)	−42 ± 3 dBV/Pa	THD@115 dB	<10%
Frequency response	100–10 kHz	THD@100 dB	<1%
S/N ratio	>55 dB	Operation Temp.	−40–85 °C
Current consumption	<200 μÅ		
